# Double bond localization in unsaturated rhamnolipid precursors 3-(3-hydroxyalkanoyloxy)alkanoic acids by liquid chromatography–mass spectrometry applying online Paternò–Büchi reaction

**DOI:** 10.1007/s00216-020-02776-5

**Published:** 2020-07-05

**Authors:** Viola Jeck, Matti Froning, Till Tiso, Lars M. Blank, Heiko Hayen

**Affiliations:** 1 Institute of Inorganic and Analytical Chemistry, University of Münster, Corrensstraße 30, 48149 Münster, Germany; 2 Present Address: Lower Saxony State Office for Consumer Protection and Food Safety – LAVES, Martin-Niemöller-Str. 2, 26133 Oldenburg, Germany; 3 iAMB – Institute of Applied Microbiology, ABBt – Aachen Biology and Biotechnology, RWTH Aachen University, 52074 Aachen, Germany

**Keywords:** Hydroxy fatty acids, Double-bond position, LC–MS, Online Paternò–Büchi reaction, GC–MS, Rhamnolipid biosurfactants

## Abstract

Lipids are biomolecules with a broad variety of chemical structures, which renders them essential not only for various biological functions but also interestingly for biotechnological applications. Rhamnolipids are microbial glycolipids with surface-active properties and are widely used biosurfactants. They are composed of one or two l-rhamnoses and up to three hydroxy fatty acids. Their biosynthetic precursors are 3-hydroxy(alkanoyloxy)alkanoic acids (HAAs). The latter are also present in cell supernatants as complex mixtures and are extensively studied for their potential to replace synthetically derived surfactants. The carbon chain lengths of HAAs determine their physical properties, such as their abilities to foam and emulsify, and their critical micelle concentration. Despite growing biotechnological interest, methods for structural elucidation are limited and often rely on hydrolysis and analysis of free hydroxy fatty acids losing the connectivity information. Therefore, a high-performance liquid chromatography-mass spectrometry method was developed for comprehensive structural characterization of intact HAAs. Information is provided on chain length and number of double bonds in each hydroxy fatty acid and their linkage by tandem mass spectrometry (MS/MS). Post-column photochemical derivatization by online Paternὸ–Büchi reaction and MS/MS fragmentation experiments generated diagnostic fragments allowing structural characterization down to the double bond position level. Furthermore, the presented experiments demonstrate a powerful approach for structure elucidation of complex lipids by tailored fragmentation.

**Electronic supplementary material:**

The online version of this article (10.1007/s00216-020-02776-5) contains supplementary material, which is available to authorized users.

## Introduction

3-(3-Hydroxyalkanoyloxy)alkanoic acids (HAAs) are known as the hydrophobic building blocks of the surface-active rhamnolipids (RLs), which are glycolipids with a hydrophilic part composed of one to two glycosidic-linked rhamnose moieties. Both the free HAAs and the glycolipids are promising alternatives to the petroleum-derived surfactants, with the latter being reported to suffer from a low biodegradation and high aquatic toxicity [1–3]. With the striving for higher environmental awareness, the microbial-derived biosurfactants are increasingly gaining attention, since they originate from renewable resources and are biodegradable [1, 4]. The glycolipid product derived from the pathogenic *Pseudomonas aeruginosa* was first described by Jarvis and Johnson in 1949 [5]. Although many other bacterial species were claimed as RL producers in recent years, *P. aeruginosa* is still regarded as the primary RL-producing microorganism [6–21].

Besides as esterified residue to the rhamnose moiety, HAAs also appear in their free form in RL extracts. Furthermore, studies explain the presence by their role as precursor for the synthesis of RLs rather than as a result of a degradation process [22]. The HAA moiety is composed of two esterified β-hydroxy fatty acids [6, 23, 24]. Moreover, with respect to variations in the chain length and the additional presence of double bonds as well as the combinatorial variety of the two β-hydroxy fatty acid chains, they present the predominate factor for the congeners’ diversity. In general, the length of hydrocarbon chains is ranging from 8 to 14 carbon atoms, with different predominant lengths depending on the RL-producing organism [6, 10, 24, 25]. Besides that, the chain can contain one to two double bonds [26, 27]. In order to vary the corresponding congeners’ properties, novel studies focus on the production of new structures divergent from the naturally synthesized, intending RLs as well the mere HAA moiety as biosurfactants [28, 29].

Nevertheless, the elucidation on double-bond level remains challenging, and corresponding reports on the pinpointing of double-bond positions in HAAs are rather limited. Furthermore, the rare studies are predominantly performed by means of nuclear magnetic resonance spectroscopy (NMR) or are GC–MS-based and thereby require preceding hydrolysis and derivatization steps. For instance, Řezanka et al. demonstrated a GC–MS method by utilizing the reaction agent dimethyl disulfide after hydrolysis of the ester bond and derivatization to the corresponding fatty acid methyl ester [26, 27]. However, these alternatives for the NMR-based approach suffer from complex preparation steps. Moreover, necessary hydrolysis steps are accompanied by the loss of structural information, especially concerning the linkage of corresponding HAA moieties.

Nevertheless, in the field of lipidomics, the overall awareness for the necessity of the structural elucidation of lipids down to the double-bond positional level is rapidly growing. This holds true especially for complex lipids like phospholipids and glycolipids. Conventional tandem mass spectrometry (MS/MS) using low-energy collision-induced dissociation (CID) is mostly insufficient for double-bond localization. Nevertheless, respective fragmentation patterns are often based on charge-directed fragmentation, yielding information on lipid class, number of carbon atoms, and degree of unsaturation of the bound fatty acid residues. As a result, a growing number of mass spectrometric approaches is reported in the literature for the challenging task of pinpointing double-bond positions in complex lipids. Reported methods are based on most varied approaches, such as charge-remote fragmentation (CRF) [30–37] and radical-induced fragmentation mechanisms [38–46] as well as ultraviolet photodissociation (UVPD) [47, 48]. One of the most straightforward approaches, based on an ozone-induced fragmentation (OzID), was implemented by Blanksby and co-workers [49, 50]. Nonetheless, OzID-based methods require a constant supply of high ozone concentrations to the mass spectrometer, which demands its modification.

**Fig. 1:**
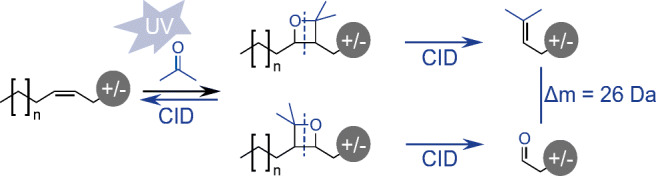
PB reaction and fragmentation scheme for the reaction of acetone with a carbon–carbon double bond (exemplified by an indicated lipid structure). Diagnostic fragments are generated with a mass difference of 26 Da

A further promising and increasingly applied mass spectrometric approach was implemented by Ma et al. in 2014, focusing on photochemical in-source functionalization [51]. The underlying name reaction, the so-called Paternὸ–Büchi (PB) reaction, is based on the UV light activation of a carbonyl group, such as acetone. As a result, the molecule with the excited carbonyl group reacts with an olefin, forming an oxetane ring, and a mixture of two site isomers per double bond is generated [52]. Furthermore, successive mass spectrometric low-energy collision-induced dissociation (CID) experiments result for each derivatized double bond in a pair of diagnostic fragments with a characteristic mass difference of 26 Da (cf. Fig. 1). Thus, these diagnostic fragments enable a precise localization of double-bond positions in lipids.

Since 2014, a number of related approaches was presented and successfully applied to a variety of biological samples [53–58]. Moreover, modifications of the initially introduced setup were reported, tackling particular drawbacks of this approach. For example, Wäldchen et al. combined the PB reaction by ultraviolet photodissociation (UVPD), striving for an even higher selectivity in terms of the fragmentation via altered dissociation process [59]. Based on a different approach, Esch et al. demonstrated the effect of charge switching, via derivatization with acetylpyridine, on the conversion to the targeted diagnostic fragments. Thus, by means of the directed cleavage in the positive ionization mode, improvements for the fragmentation pattern were reported compared to the negative ionization mode [60]. Besides that, further investigations concerning the variation of the carbonyl compound were reported with respect to targeted higher quantum yields or the suitability for tissue-based studies via matrix-assisted laser dissociation/ionization (MALDI) [61, 62].

Nonetheless, despite the diversity of the methods presented, the PB approach has primarily been applied to direct-infusion experiments. However, complex lipid mixtures lead to poor detection sensitivities, especially originating from the increased sample’s complexity after UV light exposure resulting in ion suppression effects [51, 57]. Furthermore, the shotgun approach suffers from limited structural differentiation of isomers. To tackle the latter issue, occurring from isomeric/isobaric compounds, Franklin et al. [63] demonstrated a method introduced as charge inversion, which was combined by the PB approach. As a result, the differentiation of phosphatidylcholines and phosphatidylethanolamines was attained, attributed to the differences in the reaction ability between a primary amine in the last-mentioned phospholipid class and a quaternary amine in the former phospholipid class. Nevertheless, for addressing the number of mentioned drawbacks, especially with respect to the known challenges of complex biological samples, hyphenation with LC is advantageous. Accordingly, only recently, we implemented an online approach utilizing a post-column derivatization (LC-PB-MS) [64]. Furthermore, subsequently, a second online approach was presented by Zhang et al. [65], focused on a comparable hyphenation by means of preceding HILIC separation, confirming the necessity of the chromatographic aspect. Hence, the combined approaches are increasingly recognized as essential for the improved elucidation of lipids’ double-bond positions as well as additional structural features and have to be explored further.

Subsequent to the successful hyphenation, in this work, the succeeding conversion to the essential targeted diagnostic fragments was examined more closely, since it displays a further decisive key role for the unambiguous allocation of double-bond positions, especially with respect to structurally more complex lipids. Aiming at spectra of high informative value, including abundant diagnostic fragments, the influence of the precursor ion was investigated. Hence, the effect of competing fragmentation mechanisms is discussed with respect to the two major reaction processes known as charge-driven fragmentation (CDF) and charge-remote fragmentation. Corresponding experiments are exemplified by means of the introduced structurally challenging and biotechnologically relevant HAAs.

## Materials and methods

### Chemicals and nomenclature

The HAA sample production strain *Escherichia coli* C43(DE3) carrying the *rhlA* gene from *Pseudomonas fluorescens* LMG05825 was constructed previously [29]. Furthermore, a fraction of the main congener HAA 22:1 was obtained by means of preparative reversed-phase-HPLC.

Water was purified by a Milli-Q® Academic-System (18.2 MΩ cm; 0.22 μm filter; Millipore, Molsheim, France). Lithium acetate (anhydrous, ≥ 99% for analysis) and hydrochloric acid as well as the solvents isopropanol, methanol, ACN (HPLC gradient grade), and diethyl ether (≥ 99.7%) were purchased from VWR International (Darmstadt, Germany). Acetone (HPLC grade), methyl *tert*-butyl ether (M*t*BE), boron trifluoride (BF_3_, 14% in methanol), sodium thiosulfate (≥ 99.5%), and dimethyl disulfide (≥ 99.0%) were obtained from Sigma-Aldrich (Steinheim, Germany). Ammonium acetate (≥ 99.99%) and acetic acid (≥ 99.99%) were delivered by Fluka GmbH (Darmstadt, Germany). Formic acid (99–100% p.a.) was purchased from Th. Greyer GmbH & Co. KG (Renningen, Germany). Chloroform (≥ 99.8%) and n-hexane (GC–MS grade) were obtained from Merck KGaA (Darmstadt, Germany). Sodium hydroxide (≥ 98%) and iodine were purchased from Honeywell International Inc. (Charlotte, NC, USA).

The lipids’ nomenclature was performed according to the standardized shorthand notation for lipid structures implemented by Liebisch et al. [66]. Furthermore, localized double-bond positions are indicated according to Δ nomenclature.

### Sample preparation

Purification of the aqueous supernatant from *E. coli* was carried out as described by Behrens et al. [67] to reveal the examined HAA sample. The purified HAA 22:1 sample was diluted hundredfold in ACN:H_2_O (50:50). Moreover, the photochemical direct-infusion experiments, based on the latter, were conducted by a sample diluted in acetone:H_2_O (70:30).

### Fractionation of HAAs by preparative HPLC

For the chromatographic separation, a preparative HPLC system consisting of the AZURA pump P6.1L and the AZURA autosampler 3950 (both Knauer GmbH, Berlin, Germany) connected to the SEDEX 85 LT-ELSD detector (SEDERE Olivet, France) and the fraction collector Foxy R1 (Teledyne ISCO Lincoln, USA) using a VP250/21 NUCLEODUR C18 HTec column (Macherey-Nagel GmbH & Co. KG, Düren, Germany) was employed. The flow rate was set to 10 mL/min and 3 mL sample were injected for every run. As eluent, ACN and water supplied with 0.2% (v/v) formic acid were used. The ACN concentration was linearly increased from 70% to 80% between 5 min and 35 min, and to 100% until 37 min. It was decreased back to 70% from 42 min to 52 min. The measurement was terminated after 60 min. The HAA 22:1 congener was collected between 45 min and 48 min. The ELSD detector was operated with compressed air at 3.5 bar and 40 °C. Further settings were the following: signal offset (mV), − 005; gain, 1; output signal value after AZ, + 000 mV; filter, 1S; and stray light, 100%.

### Hydrolysis and derivatization for GC–MS

For the confirmation of the HPLC–MS/MS results via gas chromatography–mass spectrometry (GC–MS), an aliquot of the preparative HPLC fraction was blown to dryness under a gentle stream of nitrogen and hydrolyzed with a 0.5 M NaOH in MeOH-H_2_O solution (9:1 *v*/*v*, 2 mL, 70 °C, 1 h). The solution was then acidified to pH 3 with 1 M HCl, and fatty acids were extracted with chloroform (3 × 3 mL). Afterwards, the solvent was removed and fatty acid methyl esters (FAMEs) were prepared by adding 100 μL of BF_3_ (14% in MeOH) and heating (75 °C, 1 h). Then, 2 mL H_2_O was added and the FAMEs extracted with chloroform (3 × 2 mL). After removal of the chloroform with a gentle stream of nitrogen, the alkylthiolation of the double bond was carried out according to the method of Shibahara et al. [68]. Therefore, 200 μL of an iodine/dimethyl disulfide solution (13 mg/mL) was added and heated (35 °C, 30 min). Then, the iodine was reduced with an aqueous Na_2_S_2_O_4_ solution (5% *w*/*v*) and 0.4 mL n-hexane/diethyl ether (1:1 *v*/*v*) was added. The organic phase was extracted, blown to dryness under a gentle stream of nitrogen, re-dissolved in 200 μL n-hexane, and used for GC–MS analysis.

### Instrumentation

#### Preliminary HPLC–MS/MS investigation

Preliminary investigations, regarding the analysis of HAA samples, were performed by means of an Agilent 1200 series HPLC system (Agilent Technologies, Santa Clara, CA, USA) coupled with a Thermo Finnigan LTQ ion trap mass spectrometer (Thermo Scientific, Bremen, Germany). The instrument was operated in the negative ionization mode using a heated electrospray source (HESI II) for ionization. Data acquisition was performed in full scan MS and MS^n^ scan mode using an isolation width of 2 Da. For a detailed description of experimental parameters of the mass spectrometric detection and the liquid chromatographic separation using an Accucore C18 column, please refer to the Electronic Supplementary Material (ESM) chapter “Preliminary HPLC-MS/MS investigation.”

Furthermore, direct-infusion experiments in the negative ionization mode, with respect to the HAA 22:1 sample, were recorded by means of the Q Exactive plus Orbitrap mass spectrometer (Thermo Fischer Scientific, Waltham, MA, USA) equipped with an electrospray ionization (ESI) probe. A syringe provided a constant flow rate of 10 μL/min. Data acquisition was performed in full-scan MS and MS/MS scan mode using an isolation width of 0.5 Da. The selected precursor ions were fragmented using higher-energy C-trap dissociation (HCD). For a detailed description of experimental parameters, please refer to the ESM chapter “Preliminary HPLC-MS/MS investigation.”

#### Photochemical activation

The online PB reaction was performed according to Jeck et al. [64] (cf. ESM Fig. S1) by means of post-column derivatization and a utilized micro-flow reactor, consisting of a deactivated fused-silica capillary and a low-pressure mercury lamp (primary emission at 254 nm, model 80-1057-01; BHK, Ontario, Canada). Prior to the reaction, the eluent flow was combined with a constant acetone flow. The latter was provided by an external syringe and adjusted to the same flow rate as the former, achieving a one-to-one dilution. The lithium-based experiments were conducted by means of an additional supply of diluted lithium acetate (0.7 mM, water-based; flow rate, 4 μL/min). With respect to a minimized system contamination, the addition was performed directly before the injection into the ionization device.

Supplementary investigations via direct-infusion were carried out at optimized reaction conditions and flow rates set to 10 μL/min. The LTQ was operated in the negative ionization mode using ESI. Data acquisition was performed in full-scan MS and MS^n^ scan mode using an isolation width of 2 Da. A detailed description of MS conditions can be found in the ESM chapter “Photochemical activation.”

#### Hyphenation with capillary HPLC

Measurements based on capillary HPLC were performed by an Agilent 1200 series capillary pump (Agilent Technologies, Waldbronn, Germany). The chromatographic separations were conducted using an Ascentis® Express C18 column (150 × 0.5 mm, 2.7 μm, Supelco®, Bellefonte, USA), and the column oven was operated at 40 °C. For experiments conducted in the positive ionization mode, by means of lithiated compounds, the eluent flow was combined by a constant flow (2 μL/min, for analysis without acetone addition) of an aqueous lithium acetate solution (0.7 mM) directly prior to the introduction into the ionization device.

Analysis of the *E. coli* extract was performed by reversed-phase capillary chromatography coupled to high-resolution MS. For a detailed description of LC–MS conditions, please refer to the ESM chapter “Hyphenation with capillary HPLC.”

Precursors for higher-energy C-trap dissociation (HCD) by the Q Exactive plus mass spectrometer were dynamically selected by means of an inclusion list, which was generated by MZmine 2.34 [69, 70] and contained a database of potential HAA species and their corresponding PB products, in a range of 16 to 32 carbon atoms and 0 to 2 double bonds in both acyl chains combined.

#### Confirmation of double-bond position by GC–MS

For GC–MS analysis, a GCMS-QP-2020 (Shimadzu, Kyoto, Japan) equipped with a Nexis GC-2030 gas chromatograph (Shimadzu, Kyoto, Japan) was used. Samples (1 μL injection volume by AOC-20i Plus autosampler) were separated on a 30-m, 0.25-mm-i.d., 0.25-μm film thickness DB-5MS column (J&W Scientific, Folsom, CA, USA). Mass spectra were obtained by electron ionization (EI, 70 eV) in the mass range *m*/*z* 50–500. Further experimental details are compiled in the ESM in chapter “GC-MS method.”

## Results and discussion

First investigations were based on the examination of chromatographic as well as fragmentation behavior of HAAs. These examinations were carried out based on a cell supernatant derived from *E. coli*, as no commercial authentic standard of HAAs is available*.* The initial investigations were conducted in the negative ionization mode, with regard to the HAAs’ proton donor properties, attributed to their free carboxyl group. However, the MS/MS experiments suffered from disadvantageous fragmentation processes driven by their structural characteristic β-hydroxy group. Therefore, further attempts were focused rather on complementary fragmentation processes by means of the so-called CRF. A successful approach by use of alkaline metal precursor ions, in this case lithiated HAAs, and an online hyphenation with the PB reaction is presented in the following.

### Chromatographic behavior and fragmentation patterns

Since HAAs mainly differ in the length of their hydrocarbon chain, their chromatography was conducted with regard to their differing hydrophobicity. Accordingly, a RP chromatography on a C18 phase was performed. In order to achieve an optimal separation, a gradient elution was applied by a mobile phase composed of ACN as organic phase and an acidified aqueous phase (5% ACN and 0.1% formic acid). As a result, a baseline separation of congeners with respect to their hydrocarbon chain length as well as their degree of unsaturation was attained. While longer chains resulted in stronger interaction with the stationary phase and longer retention times, the presence of double bonds leads to the opposite effect (cf. ESM Fig. S2). The determined sample composition was a mixture ranging from HAA 22:X to HAA 26:X, varying between no and one double bond (i.e., *X* = 0 or *X* = 1). Moreover, the chromatography revealed the presence of isomeric compounds by displaying additional baseline separated peaks with the same *m*/*z* or prominent peak shoulders.

**Fig. 2:**
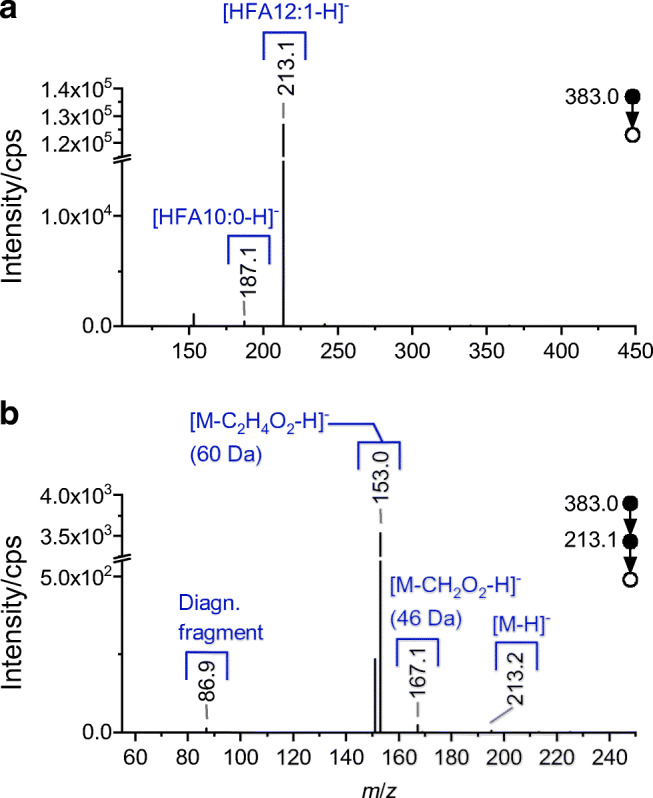
LC–MS/MS spectrum in the negative electrospray ionization mode of HAA 22:1 with precursor *m*/*z* 383 ([M−H]^−^) and retention time 8.83–9.49 min. Mass spectra of HAA 22:1 (corresponding to HAA 12:1/10:0). Displayed is the MS/MS (**a**) and subsequent MS^3^ spectrum of HFA 12:1 (**b**), with indicated diagnostic fragment for the double bond in position Δ 5 (cf. Fig. 3b)

For gaining information on the HFA level, fragmentation experiments were conducted. The characteristic fragmentation pattern obtained in the negative ionization mode is exemplified by HAA 22:1 and displayed in Fig. 2 and ESM Fig. S3 for the proposed two isomers, eluting after 8.3 and 9.1 min, respectively.

**Fig. 3:**
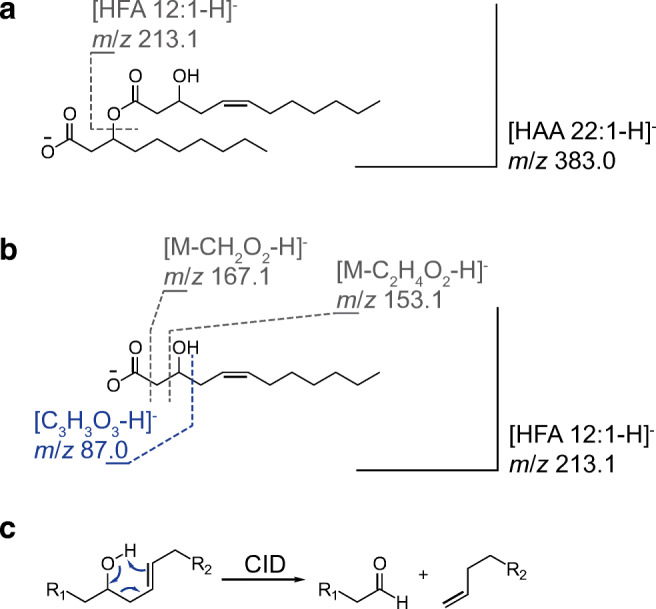
Fragmentation pattern of examined HAA 22:1 (corresponding to HAA 12:1/10:0), conducted in the negative ionization mode ([M−H]^−^) (**a**). Subsequent obtained fragmentation pattern of HFA 12:1, with indicated diagnostic fragment *m*/*z* 87.0 (**b**). Proposed charge-remote allylic fragmentation (R_1_ and R_2_ representing the carboxyl group and the terminal end of the hydrocarbon chain, respectively) (**c**)

The obtained MS/MS spectra displayed in Fig. 2 and in ESM Fig. S3 show different fragments and can thus be allocated to different HFA combinations. For example, the later eluting and abundant peak indicates fragments that can be allocated to HFA 10:0 and HFA 12:1. According to Lépine et al. [22], the fragments solely derive from the cleaved terminal HFAs at the hydroxyl end. Hence, the fragments can be attributed to two different isomers HAA C_10:0_/C_12:1_ vs. HAA C_12:1_/C_10:0_ with the terminal HFA interchanged (cf. Fig. 3a, indicating the fragmentation).

The earlier eluting low abundant peak, therefore, can be allocated to two isomeric compounds composed of HFA 8:0 and HFA 14:1 (HAA C_8:0_/C_14:1_ vs. HAA C_14:1_/C_8:0_). Accordingly, the HAAs with the shorter saturated HFA chain (HFA 8:0) have shorter retention times than those with the larger saturated HFA chain (HFA 10:0) due to reduced hydrophobic interaction with the stationary phase.

Interestingly, a subsequent fragmentation of the generated HFA product ions revealed further distinctive structural information. The fragmentation of [HFA 12:1-H]^−^ resulted, on the one hand, in diagnostic cleavages at the polar carboxylic side that point to the hydroxylation in the β position (*m*/*z* 167.1 and 153.0; cf. Fig. 2b). Corresponding proposed product ion structures are consistent with previous CID fragmentation experiments on HFAs investigated by Marshall et al. [71]. On the other hand, an additional low abundant fragment at *m*/*z* 86.9 was obtained. The fragment is assumed to result from a doubly activated cleavage, which is attributed to a double bond located in position Δ 5 (see Fig. 3b). More precisely, the carbon–carbon bond cleavage is facilitated by the bond’s beneficial position adjacent to the hydroxy group and allylic to the double bond. The particular dissociation is assumed to involve a charge-remote allylic fragmentation as suggested for comparable experiments demonstrated in the literature [72, 73]. In comparison, the equivalent spectra generated from [HFA 12:0-H]^−^ (cf. ESM Fig. S4) merely display mentioned cleavages attributed to the β-hydroxy group. Moreover, fragmentation experiments that involved different unsaturated HFAs with divergent hydrocarbon chain length suffered from the same lack of diagnostic fragments. Both experiments, however, confirm the distinctive diagnostic fragment originating from the double-bond position Δ 5. Moreover, the fragmentation patterns of the further examined HFAs, reversely, indicate a divergent double-bond position. For verification, subsequent investigations by means of HRMS and direct-infusion setup were conducted, which confirmed the assumption via accurate masses (e.g., *m*/*z* 87.0088, C_3_H_3_O_3_^−^, Δ *m*/*z* = 0.4 ppm).

### Capillary flow rate and PB-LC-MS

#### PB direct- infusion

**Fig. 4:**
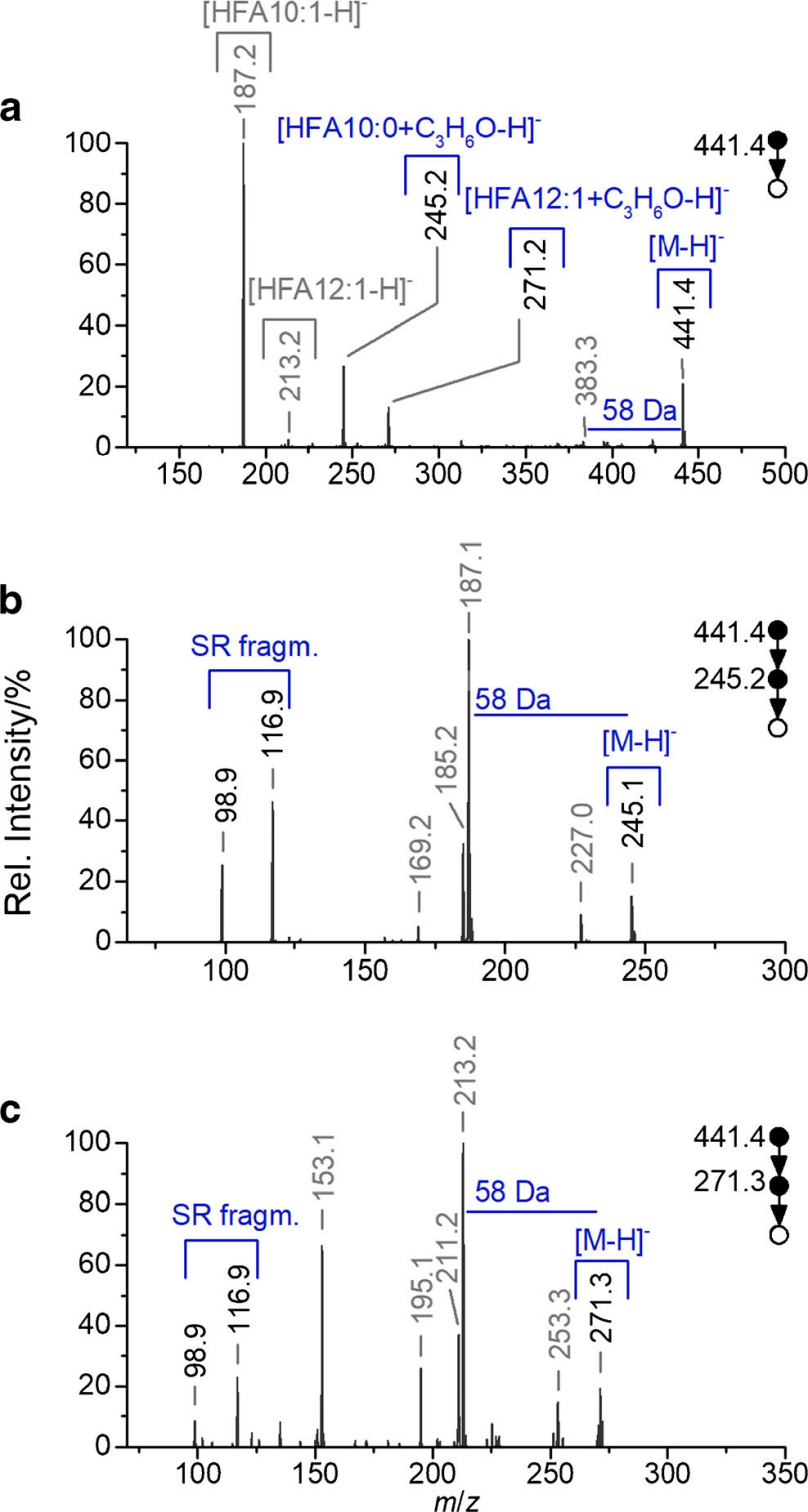
Spectra received from derivatized HAA 22:1, obtained via direct-infusion and micro-flow reaction, conducted in the negative ionization mode ([M−H]^−^). Displayed is the MS/MS (**a**) and subsequent MS^3^ spectrum of the derivatized HFA 12:1 (**b**) as well as the derivatized HFA 10:0 (**c**). Indicated are the acetone losses (58 Da) and fragments received for a dominant side reaction (SR fragm.; cf. Fig. 5)

In order to establish a method that pinpoints double bonds, regardless of their particular position in HAAs, further investigations were based on the application of a LC–PB–MS hyphenation, introduced by Jeck et al. [64]. However, prior to the hyphenation experiments, pretests were conducted via direct-infusion experiments and micro-flow reaction. Therefore, a purified and enriched fraction of HAA 22:1 was used which has been obtained by preparative HPLC (see section “Materials and methods”). The resulting mass spectra of HAA 22:1, recorded in the negative ionization mode, are displayed in Fig. 4.

**Fig. 5:**
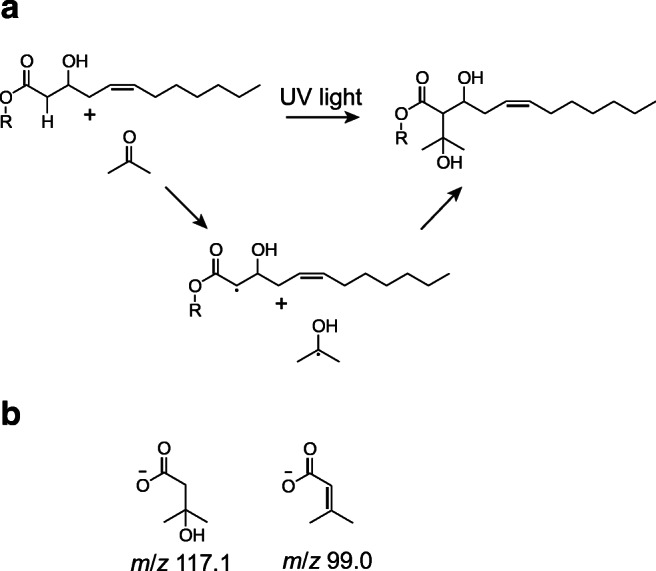
Postulated side reaction, attributed to a hydrogen abstraction in the α position by an excited acetone, the latter yielding a 2-hydroxy-2-propyl radical [75, 76], and a subsequent radical recombination. Displayed is the suggested reaction scheme, with R corresponding to the attached HFA 10:0 residue (**a**) as well as the obtained characteristic fragments (**b**)

According to previous experiments, acetone addition to the targeted HAA was observed as a response to the applied UV light irradiation. However, the MS/MS spectrum indicates an acetone addition to both HFA moieties (*m*/*z* 245.2 and 271.2), regardless of the existence of a double bond. Furthermore, no diagnostic fragments resulting from the derivatized HFA 12:1 were obtained (cf. Fig. 4c). Especially the former aspect suggests a competing addition reaction, which is independent of the presence of a double bond. One of the main known side reactions, occurring in the course of the PB reaction, is the hydrogen abstraction at the allylic position of the double bond. The reaction is a result of the labile hydrogen atom and the relatively stable radical intermediate [52, 74]. HAAs, however, possess a further labile hydrogen atom (α position), which is attributed to its hydroxylation in the β position. Furthermore, a hydrogen abstraction by the excited acetone produces the well-known 2-hydroxy-2-propyl radical [75, 76]. A subsequent radical recombination, lastly, forms the suggested side product (see Fig. 5a). Interestingly, further evidence for the proposed conversion is given by the obtained MS^3^ spectra. Alongside the fragments indicating an acetone loss, two further characteristic fragments at *m*/*z* 98.9 and 116.9 were obtained, which can be explained by a cleavage between the α and β position (see Fig. 4b, c). Furthermore, the fragmentation of the derivatized double bond appears to be disadvantageous compared to further obtained unspecific fragmentation processes. Hence, no diagnostic fragments were observed for pinpointing the position. Based on this, further attempts were focused on a complementary fragmentation approach directed by lithiated precursor ions. Since alkaline metals are known to promote CRFs, an improvement of the fragmentation pattern was expected. In accordance with that, the conducted experiments resulted in a significant reduction of unspecific fragments originating from a CDF mechanism. Moreover, abundant signals for the essential diagnostic fragments were obtained, which will be discussed in detail below.

#### LC–PB–MS

**Fig. 6:**
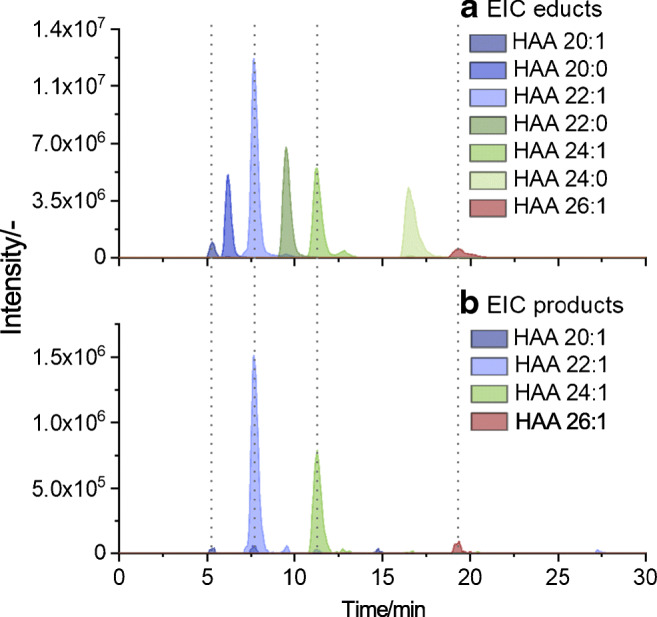
Obtained chromatographic separation for HAA congeners of the cell supernatant derived from *E. coli*, conducted by means of LC–PB–MS and recorded in the positive ionization mode ([M+Li]^+^). Illustrated is the correlation of peaks corresponding to unsaturated HAAs (**a**) and their corresponding derivatized counterparts (**b**)

**Fig. 7:**
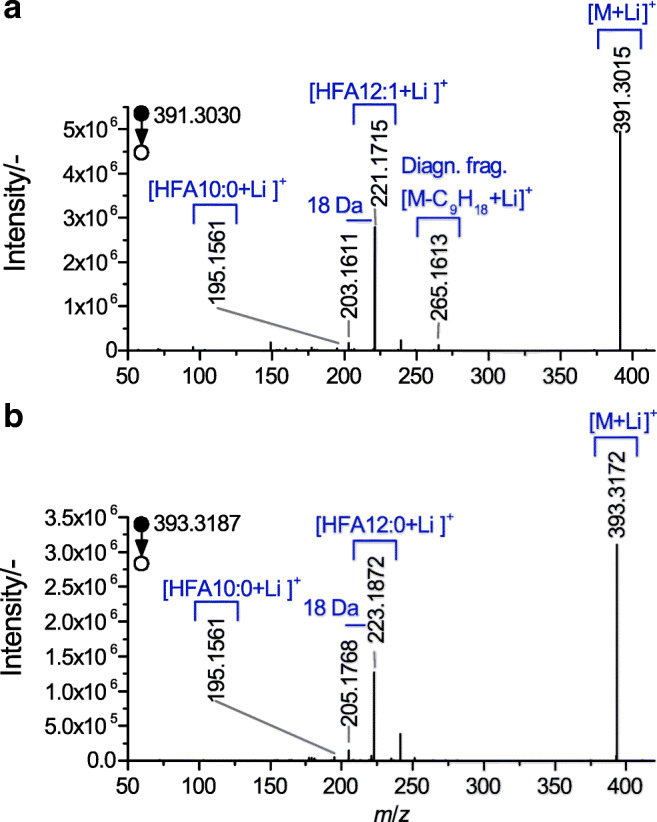
MS/MS spectra received from HAA 22:1 (7.35–8.21 min) (**a**) and HAA 22:0 (9.18–10.03 min) (**b**), obtained via LC–PB–MS, conducted in the positive ionization mode ([M+Li]^+^). Displayed is the characteristic fragmentation pattern, with the indicated diagnostic fragment for the former (*m*/*z* 265.1613; cf. Fig. 8)

With regard to the online hyphenation by means of the LC–PB–MS approach, the initial chromatographic separation was transferred to capillary flow rates. The HPLC flow rate was set to 13 μL/min and combined, directly prior to the introduction to the ESI probe, by an additional flow of an aqueous lithium acetate solution (2 μL/min, for analysis without acetone addition). The applied gradient was, compared to the previous one, adjusted to a steeper increase of the organic content over time. Thus, the large dwell volume of the utilized capillary pump was compensated to a certain degree although it was accompanied by a loss of separation efficiency. Moreover, the hyphenation by the micro-flow reactor resulted in further moderate peak broadening. Nevertheless, a sufficient separation was attained, which presents a baseline separation for HAA congeners of different carbon numbers and degrees of unsaturation, which prevents interferences by ^13^C isotopes. In addition, isomeric structures could be differentiated by partly chromatographically separated peaks. Based on attained separation, the LC–PB–MS measurements were performed with a reaction time of 7 s. The particular correlation of peaks corresponding to unsaturated HAAs and their respective derivatization products is demonstrated in Fig. 6. The recorded characteristic MS/MS spectra, obtained for the HAAs and their PB products, are displayed in Fig. 7 and Fig. 9, exemplified by HAA 22:0 and HAA 22:1, respectively.

**Fig. 8:**
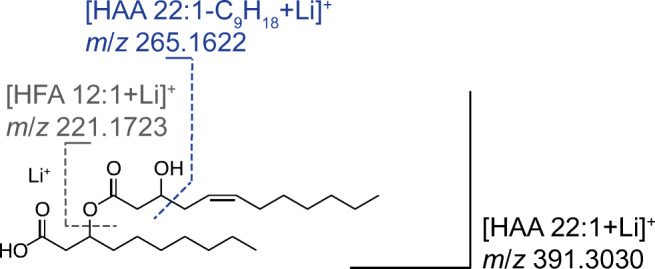
Fragmentation pattern of examined HAA 22:1 (corresponding to HAA 12:1/10:0), conducted in the positive ionization mode ([M+Li]^+^), with indicated diagnostic fragment *m*/*z* 265.1622

With respect to the educts, similarities can be observed by comparing the MS/MS spectra with the spectra conducted in the negative ionization mode. Both approaches resulted in abundant product ions that can be assigned to the terminal HFA at the hydroxyl end. However, in the case of the lithiated precursor ion, additional low-abundant fragments can be observed, e.g., attributed to a subsequent water loss. More interestingly, the fragmentation of [HAA 22:1 + Li]^+^ leads to the generation of an additional signal at *m*/*z* 265.1613. The product ion can be explained by a cleavage induced by the double bond in the position Δ 5. Furthermore, the fragment confirms the suggested origin of the previously discussed *m*/*z* 86.9, which was obtained in the negative ionization mode after a subsequent fragmentation of HFA 12:1. By means of the Li^+^ coordinated precursor ion, however, the underlying CRF is assumed to be promoted. Hence, the cleavage occurs without an additional fragmentation step and, furthermore, the preceding scission of the HFA moieties (cf. fragmentation scheme in Fig. 8).

**Fig. 9:**
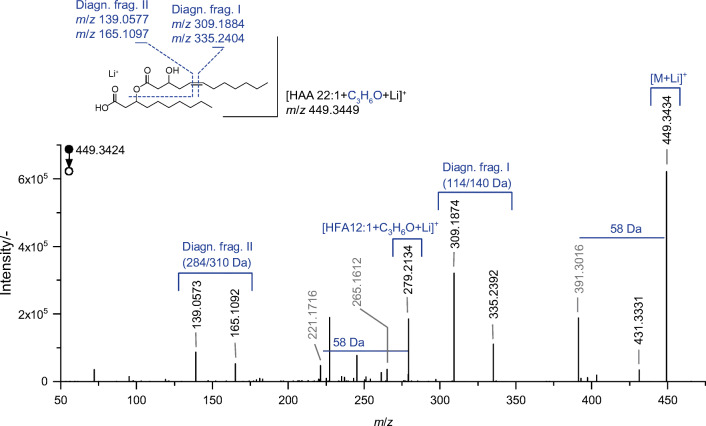
MS/MS spectrum received from derivatized HAA 22:1 (corresponding to HAA 12:1/10:0; 7.69 min) obtained via LC–PB–MS, conducted in the positive ionization mode ([M+Li]^+^). Displayed is the characteristic fragmentation pattern, with indicated acetone loss (58 Da) and pairs of diagnostic fragments (diagn. frag. I/II)

Besides that, a significant improvement was observed regarding the fragmentation pattern of the PB products. By means of the Li^+^ coordination, abundant signals were obtained which can be allocated to the cleavage of the derivatized double bonds. Two sets of diagnostic fragments were generated enabling the unambiguous pinpointing of double-bond positions. The generated fragments resulted either from the sole cleavage of the oxetane ring or an additional subsequent loss of the esterified second HFA moiety. Consequently, a further confirmation of the positional information is given, as well as a distinct assignment to a particular HFA moiety. The derivatized HAA 22:1, for example, resulted in the two diagnostic pairs of fragments at *m*/*z* 309.1874 and 335.2392 as well as *m*/*z* 139.0573 and 165.1092 (cf. Fig. 9). The first pair indicated neutral losses that can be assigned to a ω 7 double bond. However, due to the second pair, a loss of a HFA 10:0 moiety is pointed out, which enabled the distinct allocation to a Δ 5 position of a HFA 12:1 moiety. Moreover, further confirmation about the structural arrangement of the two HFA moieties is provided by additional signals, which are comparable to the pattern received form the educt.

Noteworthily, the results for HAA 22:1 are in accordance with the previous suggested double-bond position by means of the fragments *m*/*z* 86.9 (LTQ) and 265.1613 (Q Exactive plus), received from the non-derivatized precursor ion in the negative and positive ionization mode, respectively. Interestingly, the latter fragment was also obtained by means of the lithiated derivatized precursor ion (*m*/*z* 265.1612).

According to the same pattern, the further congeners of the mixture were examined concerning their HFA composition and double-bond position. The results are listed in Table 1. With respect to the Δ nomenclature, double-bond positions ranging from Δ 5 to 9 were unambiguously discovered. However, applying the ω nomenclature, the variance of the HAAs’ double-bond position showed to be limited to the ω 7 position, which represents a known double-bond position for HAAs derived from strains such as *P. aeruginosa*. Only the spectrum obtained for HAA 10:1/10:0 suffered from limited informative value, preventing a localization, which is attributed to its very low abundance and, furthermore, the coelution with HAA 12:1/8:0. Nevertheless, obtained data is consistent with previous reports for HAAs derived from a related species, *Pseudomonas aeruginosa* [27, 77].

**Table 1:** Identified HAA congeners in a cell supernatant of *E. coli*, obtained by means of LC–PB–MS, conducted by lithiated precursor ([M+Li]^+^)

Accuratemass *m*/*z*	δm/m[ppm]	Intensity (height ofchromatogram)	Retentiontime[min]	Congener	Chaincombination^a^	PB product/precursor *m*/*z*	Diagnostic fragments *m*/*z*	Double-bondposition
363.2705	− 3.4	1.05e6	5.33	20:1	**12:1**/8:0; 8:0/**12:1**	421.3104	281.1567 + 307.2077; 139.0573 + 165.1094	ω7/Δ5
363.2705	− 3.4	6.75e4	5.79	20:1	**10:1**/10:0; 10:0/**10:1**	421.3104	281.1567 + 307.2077	ω7/Δ3^c^
365.2863	− 3.0	5.85e6	6.20	20:0	10:0/10:0	/	/	/
365.2863	− 3.0	/^b^	6.33	20:0	12:0/8:0; 8:0/12:0	/	/	/
391.3015	− 3.9	5.43e5	7.25	22:1	**14:1**/8:0; 8:0/**14:1**	449.3429	309.1873 + 335.2392; 167.0896 + 193.1286	ω7/Δ7
391.3015	− 3.9	1.36e7	7.68	22:1	**12:1**/10:0 10:0/**12:1**	449.3429	309.1873 + 335.2392; 139.0573 + 165.1092	ω7/Δ5
393.3172	− 3.8	7.23e6	9.52	22:0	12:0/10:0; 10:0/12:0	/	/	/
393.3172	− 3.8	/^b^	9.94	22:0	14:0/8:0; 8:0/14:0	/	/	/
419.3329	− 3.4	6.11e6	11.22	24:1	**14:1**/10:0; 10:0/**14:1**	477.3737	337.2185 + 363.2705; 167.0885 + 193.1406	ω7/Δ7
419.3330	− 3.2	5.05e5	12.86	24:1	**12:1**/12:0; 12:0/**12:1**	477.3737	337.2184 + 363.2704; 139.0572 + 165.1093	ω7/Δ5
421.3485	− 3.5	/^b^	16.34	24:0	12:0/12:0	/	/	/
421.3485	− 3.5	4.64e6	16.50	24:0	14:0/10:0; 10:0/14:0	/	/	/
447.3642	− 3.2	6.28e5	19.25	26:1	**16:1**/10:0; 10:0/**16:1**	505.4042	365.2499 + 391.3017; 195.1198 + 221.1714	ω7/Δ9
447.3641	− 3.4	2.71e5	20.12	26:1	**14:1**/12:0	505.4042	365.2499 + 391.3011	ω7/Δ7

^a^Unsaturated hydroxy fatty acid residues are highlighted in bold

^b^No information provided attributed to coelution with isomeric congener

^c^Unambiguous annotation of double-bond position not possible due to low abundance as well as coelution

The approach by means of lithiated precursor ions proved to be valuable for the structural elucidation of HAAs, especially with respect to the pinpointing of double bonds. Although the structure rather suggests an ionization conducted in the negative ionization mode, for yielding the highest ionization efficiencies, the success of the LC–PB–MS approach appeared to rather depend on the precursor ion.

#### GC–MS results

The assignment of double-bond positions in HAAs by LC–PB–MS was confirmed by an independent method, i.e., GC–MS. Similar to Kendel et al. and Řezanka et al., we performed GC–MS after hydrolysis of the ester bond and a double derivatization strategy [26, 27, 78]. First, corresponding fatty acid methyl esters (FAMEs) were formed, and second, double bonds were reacted with dimethyl disulfide. Electron ionization (EI) of the derivatives yielded diagnostic fragments to pinpoint the double-bond position. For the derivative of HFA 12:1, the ions at *m*/*z* 145 and at *m*/*z* 177 indicate the position of the double bond as ω7/Δ5 (cf. ESM Fig. S5). Further characteristic ions are *m*/*z* 74 (McLafferty rearrangement) and *m*/*z* 103 (α cleavage next to the hydroxyl group) and confirm the position of the hydroxyl group as 3-OH.

In comparison to the presented LC–PB–MS method, pinpointing of double-bond positions by GC–MS required a hydrolysis step which is accompanied by the loss of structural information, especially concerning the linkage of corresponding HAA moieties. Therefore, the here presented method based on online PB reaction provides a more comprehensive picture of HAAs’ structure.

## Conclusions

Subsequent to the successful hyphenation of high-performance liquid chromatography (HPLC) with the online Paternò–Büchi (PB) reaction, as presented previously [64], this work focused on the just as important and challenging successive conversion to targeted diagnostic fragments. This conversion was explored by a comparison of different precursor ions with respect to the known major reaction processes, classified as charge-driven fragmentation (CDF) and charge-remote fragmentation (CRF) reaction [79, 80, 81]. As expected, a gain of control concerning the spectra’s information value, and thus the abundance of diagnostic fragments, was achieved. However, the investigations carried out on the structurally challenging lipid class of HAAs demonstrated the limitations of the approach performed by deprotonated precursor ions. Furthermore, it presented the possibilities of lithium-coordinated precursor ions. Initial investigations were examined in the negative ionization mode, with respect to the HAAs’ acidic and, thus, proton donor properties. However, the fragmentation experiments suffered from disadvantageous cleavages originating from the structural characteristic β-hydroxy group. Consequently, diagnostic fragments were neither generated by means of single nor multiple fragmentation experiments. Interestingly, fragmentation experiments based on the mere underivatized HAA 22:1 resulted, in comparison, in a distinct product ion induced by the double bond in the Δ 5 position. However, this particular fragmentation and thus the elucidation on the double-bond positional level was solely observed for the specific position. The specific carbon–carbon bond cleavage is assumed to be doubly activated by the bond’s beneficial position adjacent to the hydroxy group and allylic to the double bond. Nevertheless, subsequent experiments were conducted by means of lithiated precursor ions, leading to spectra of higher informative value, including abundant diagnostic fragments. Based on this, the analysis of the cell supernatant of a recombinant *Escherichia coli* strain carrying the gene for the acyltransferase RhlA from *P. fluorescens* LMG05825 was successfully conducted by means of LC–PB–MS, with respect to the HAAs’ congener composition and its detailed structural elucidation. Furthermore, the proven gain of control concerning the spectra’s informational value, demonstrated by HAAs as model compounds, presents a powerful tool and can equally be applied to the elucidation of further complex lipid classes.

## Electronic supplementary material

ESM 1 (PDF 522 kb)
