# Supplementary material for: Double bond localization in unsaturated rhamnolipid precursors 3-(3-hydroxyalkanoyloxy)alkanoic acids by liquid chromatography–mass spectrometry applying online Paternò–Büchi reaction
Source: Anal Bioanal Chem. 2020 Jul 5;412(23):5601–13. doi: 10.1007/s00216-020-02776-5 (PMC7413879; doi:10.1007/s00216-020-02776-5)
Supplement: Supplementary file 1 — (PDF 522 kb) [file 216_2020_2776_MOESM1_ESM.pdf]

Analytical and Bioanalytical Chemistry

Electronic Supplementary Material

Double bond localization in unsaturated rhamnolipid precursors 3-(3-hydroxyalkanoyloxy)alkanoic acids by liquid chromatography-mass spectrometry applying online Paternò-Büchi reaction

Viola Jeck, Matti Froning, Till Tiso, Lars M. Blank, Heiko Hayen

Abstract

Additional information on the instrumental set-up of the online Paternò-Büchi reaction is provided (cf. Fig. S1). Furthermore, LC-MS^n (cf. Figs. S2, S3, S4) and GC-MS data (cf. Fig. S5) for the localization of double bond positions are presented.

Photochemical activation

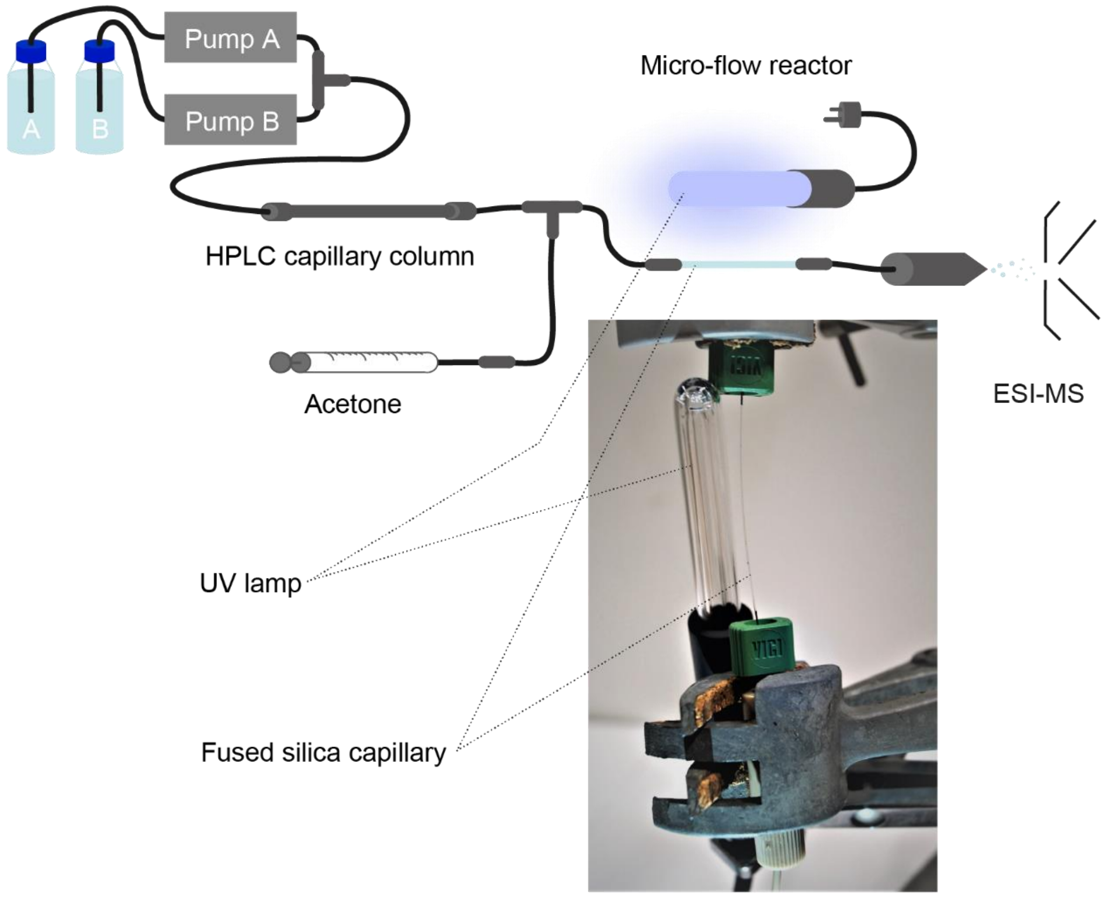

Fig. S1 Schematic setup showing the hyphenation of online PB reaction with HPLC

The online PB reaction was performed (cf. Fig. S1) by means of post-column derivatization and a utilized micro-flow reactor, consisting of a deactivated fused-silica capillary and a low-pressure mercury lamp (primary emission at 254 nm, model 80-1057-01; BHK, Ontario, Canada). Prior to the reaction, the eluent flow was combined with a constant acetone flow. The latter was provided by an external syringe and adjusted to the same flow rate as the former, achieving a one-to-one dilution. The lithium-based experiments were conducted by means of an additional supply of diluted lithium acetate (0.7 mM, water-based; flow rate 4 µL/min). With respect to a minimized system contamination, the addition was performed directly before the injection into the ionization device.

Supplementary investigations via direct-infusion were carried out at optimized reaction conditions and flow rates set to 10 µL/min. The Thermo Finnigan **LTQ ion trap mass spectrometer** (Thermo Scientific, Bremen, Germany) was operated in the negative ionization mode using ESI. Data acquisition was performed in full scan MS and MS^n scan mode using an isolation width of 2 Da. The selected precursor ions were fragmented with normalized collision energy at 15% for MS^2 and 20% for MS^3 spectra. The automatic gain control (AGC) target was set to 3000 with a maximum injection time of 10 ms for full time spectra and to 1000 with a maximum injection time of 100 ms for MS^n spectra. The source voltage was set to 2.5 kV with a sheath gas (nitrogen) flow rate of 6 AU and an auxiliary gas (nitrogen) flow rate of 4 AU. The capillary temperature was set to 275 °C.

Hyphenation with capillary HPLC

Measurements based on capillary HPLC were performed by an Agilent 1200 series capillary pump (Agilent Technologies, Waldbronn, Germany). The chromatographic separations were conducted using an Ascentis® Express C18 column (150 x 0.5 mm, 2.7 µm, Supelco®, Bellefonte, USA) and the column oven was operated at 40°C. For experiments conducted in the positive ionization mode, by means of lithiated compounds, the eluent flow was combined by a constant flow (2 µL/min, for analysis without acetone addition) of an aqueous lithium acetate solution (0.7 mM) directly prior to the introduction into the ionization device.

For the separation of the *E. coli* extract, a mobile phase composed of 10 mM NH_4Ac buffer pH 5.75, 5% ACN (phase A) and ACN (phase B) was utilized. The gradient was set up as follows: 0 - 0.1 min, 70% B; 0.1 – 10 min, from 70 to 90% B, and hold at 90% until 55 min; then back to 70% for equilibration of the column. The flow rate was set to 13 µL/min and an aliquot of 2 µL sample was injected.

For data acquisition a **Q Exactive plus**, equipped with an HESI II source and operated in the positive ionization mode, was utilized. The full scan MS/ data-dependent-MS/MS mode was performed with an isolation width of 1 Da. The selected precursor ions were fragmented using higher energy C-Trap dissociation (HCD) with normalized collision energy of 25%. The resolution was set to 70,000 (full width at half-maximum (fwhm) at *m*/*z* = 200) for full scan profile spectra and to 35,000 (fwhm at *m*/*z* = 200) for centroid MS/MS spectra. The AGC target was set to 1 x 10^6 with a maximum injection time of 100 ms for full scan spectra and to 1 x 10^5 with a maximum injection time of 50 ms for MS/MS spectra. The source voltage was set to 3.5 kV with a sheath gas (nitrogen) flow rate of 17 AU and an auxiliary gas (nitrogen) flow rate of 6 AU. The capillary temperature was set to 250 °C while the source temperature was set to 60 °C.

Preliminary HPLC-MS/MS investigation

Preliminary investigations, regarding the analysis of HAA samples were performed by means of an Agilent 1200 series HPLC system (Agilent Technologies, Santa Clara, CA, USA) coupled with a Thermo Finnigan **LTQ ion trap mass spectrometer**. The instrument was operated in the negative ionization mode using a heated electrospray source (HESI II) for ionization. Data acquisition was performed in full scan MS and MS^n scan mode using an isolation width of 2 Da. The selected precursor ions were fragmented with normalized collision energy of 35%. The automatic gain control (AGC) target was set to 3000 with a maximum injection time of 10 ms for full scan spectra and to 1000 with a maximum injection time of 100 ms for MS^n spectra. The source voltage was set to 3 kV with a sheath gas (nitrogen) flow rate of 20 arbitrary units (AU) and an auxiliary gas (nitrogen) flow rate of 15 AU. The capillary temperature was set to 330 °C, while the source heater temperature was adjusted to 60 °C.

For the chromatographic separation, an Accucore C18 column (150 x 2.1 mm, 2.6 µm, Thermo Fischer Scientific, Waltham, MA, USA) was utilized and the temperature of the column heater was set to 40°C. Investigations were conducted by injection of 10 µL and a set flow rate of 0.25 mL/min. The mobile phases were composed of H_2O, 5% ACN and 0.1% formic acid (phase A) and ACN (phase B). The gradient was as follows: 0 - 1 min, 70% B; 1 - 30 min, from 70 to 100% B, and hold at 100% B until 35 min; then back to 70% B and hold until 45 min for equilibration of the column.

Furthermore, direct-infusion experiments in the negative ionization mode, with respect to the HAA 22:1 sample, were recorded by means of the **Q Exactive plus Orbitrap mass spectrometer** (Thermo Fischer Scientific, Waltham, MA, USA) equipped with an electrospray ionization (ESI) probe. A syringe provided a constant flow rate of 10 µL/min. Data acquisition was performed in full scan MS and MS/MS scan mode using an isolation width of 0.5 Da. The selected precursor ions were fragmented using higher energy C-Trap dissociation (HCD) with normalized collision energy at 30% and in source fragmentation with normalized collision energy at 35 eV. The resolution was set to 280,000 (full width at half-maximum (fwhm) at *m/z* = 200). The AGC target was set to 1 x 106 with a maximum injection time of 50 ms. The source voltage was set to 3 kV with a sheath gas (nitrogen) flow rate of 15 AU and an auxiliary gas (nitrogen) flow rate of 5 AU. The capillary temperature was set to 300 °C.

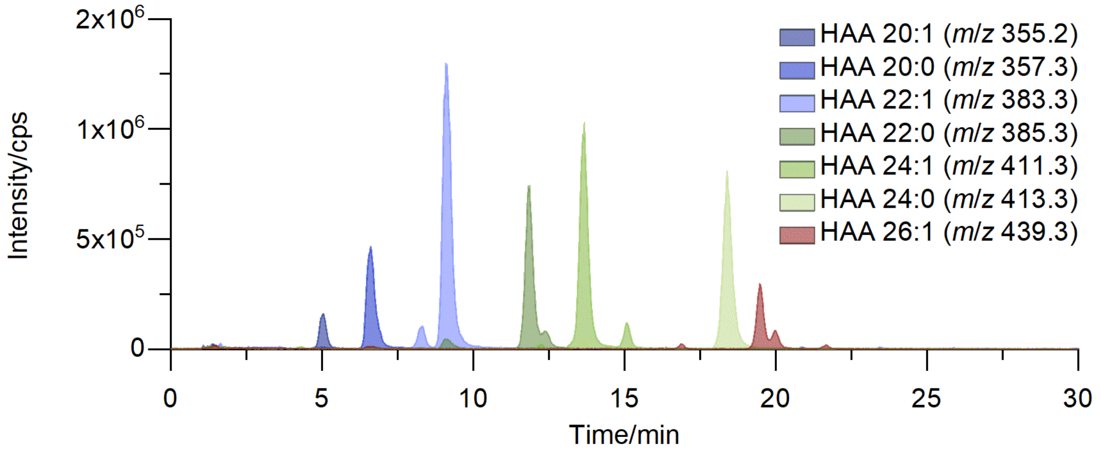

Fig. S2 Chromatographic separation of HAA congeners in an E. coli cell supernatant, conducted by means of LC-MS (narrow bore column) and recorded in the negative electrospray ionization mode ($[M-H]^-$)

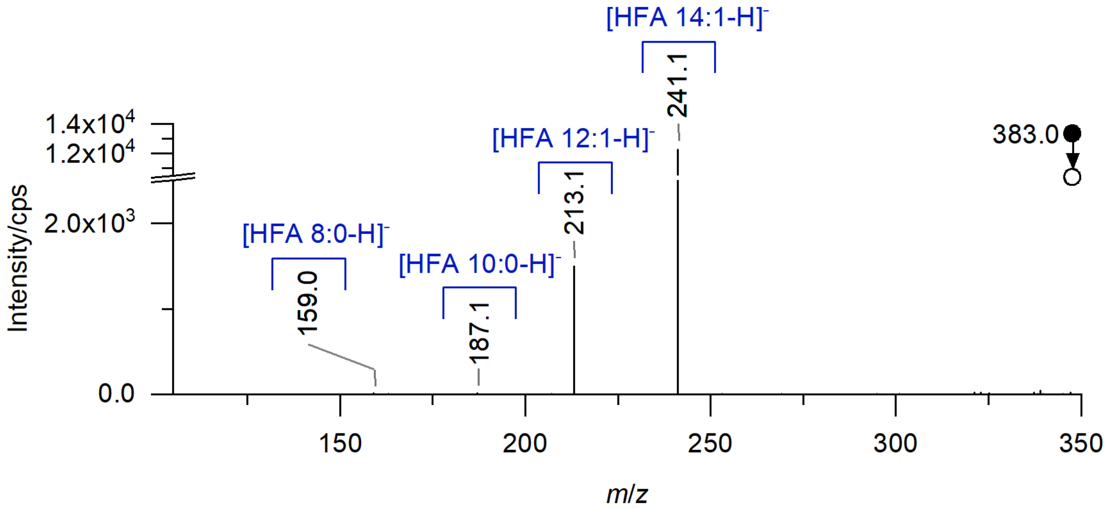

Fig. S3 LC-MS/MS spectrum in the negative electrospray ionization mode of HAA 22:1 with precursor *m/z* 383 ($[M-H]^-$) and retention time 7.90-8.52 min. Two hydroxy fatty acid combinations were detected, HAA 14:1/8:0; and HAA 12:1/10, respectively

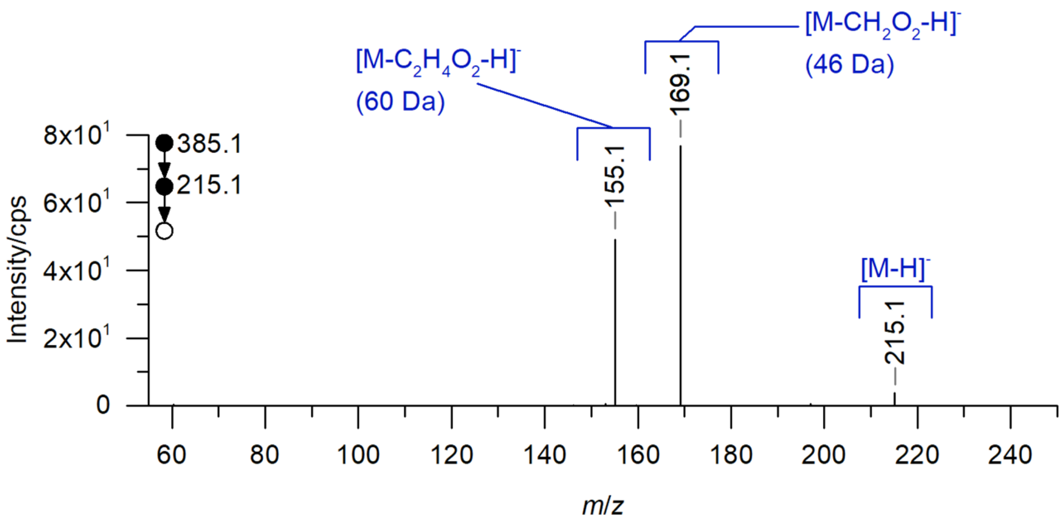

Fig. S4 LC-MS^3 spectrum of HAA 22:0 (*m/z* 385.1, corresponding to HAA 12:0/10:0; 11.31-12.27 min) and the subsequent MS^3 fragmentation of HFA 12:0 (*m/z* 215.1), obtained by means of LC-MS and conducted in the negative ionization mode ($[M-H]^-$). The neutral mass losses from precursor *m/z* 215.1 are indicated in brackets

GC-MS method

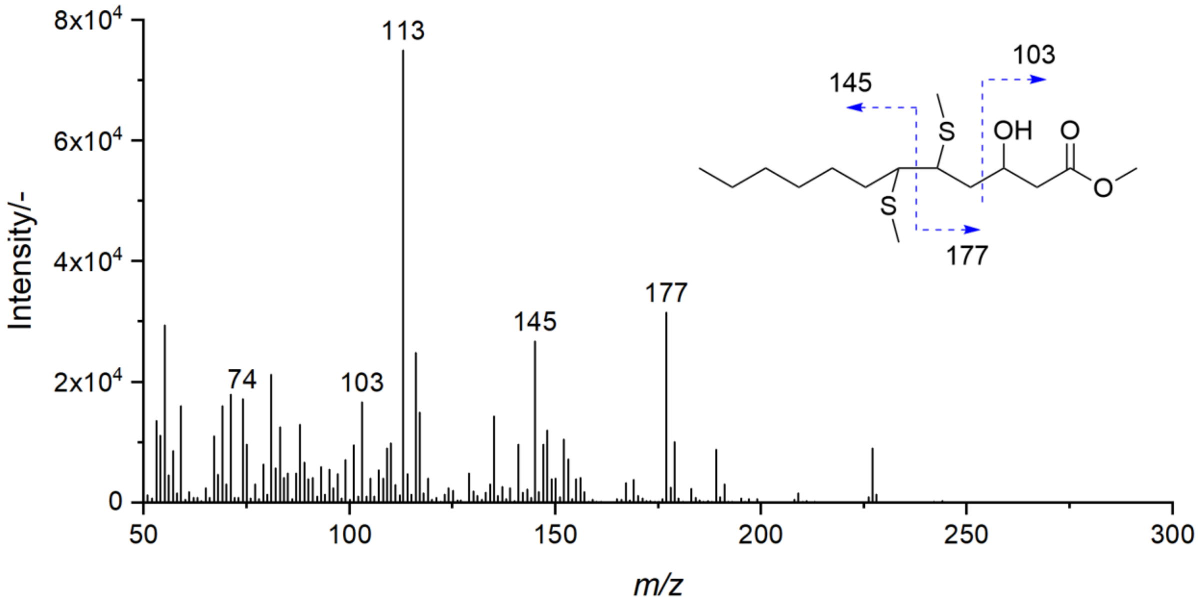

Fig. S5 GC-MS spectrum of HFA 12:1 after methylation and alkylthiolation. The ions at *m/z* 145 and *m/z* 177 are formed by cleavage of the activated double bond and indicate the double bond position. According to the LC-MS/MS results the double bond position is identified as ω7/Δ5. The ion with *m/z* 103 results from α cleavage next to the hydroxyl group and indicates its position (3-OH). The *m/z* 74 fragment results from the McLafferty rearrangement at the carboxyl end

For GC-MS analysis a **GCMS-QP-2020** (Shimadzu, Kyoto, Japan) equipped with a Nexis GC-2030 gas chromatograph (Shimadzu, Kyoto, Japan) was used. Samples were separated on a 30 m, 0.25 mm i.d., 0.25 µm film thickness DB-5MS column (J&W Scientific, Folsom, California, United States of America). Using an AOC-20i Plus autosampler (Shimadzu, Kyoto,

Japan) and PTV inlet (250 °C) in splitless mode, samples (1 µL) were introduced into the system. Helium (5.0) was used as carrier gas with a flow rate of 1.16 mL/min. The column oven was programmed as follows: Starting at 100 °C, the temperature was increased at a rate of 5 °C/min to 300 °C, which was held for 20 min. Mass spectra were obtained by electron ionization (EI, 70 eV). The temperature of the ion source and interface were set to 250 °C. Data were recorded from *m/z* 50-500 with a rate of 10 scans/s. For comparison a 2-Hydroxy-9-octadecenoic acid standard (≥98%, Sigma Aldrich, Steinheim, Germany) was hydrolyzed and derivatized as described above.
